# Prophylaxis and Treatment of Alzheimer's Disease by Delivery of an Adeno-Associated Virus Encoding a Monoclonal Antibody Targeting the Amyloid Beta Protein

**DOI:** 10.1371/journal.pone.0057606

**Published:** 2013-03-28

**Authors:** Masaru Shimada, Shinya Abe, Toru Takahashi, Kazumasa Shiozaki, Mitsue Okuda, Hiroaki Mizukami, Dennis M. Klinman, Keiya Ozawa, Kenji Okuda

**Affiliations:** 1 Department of Molecular Biodefense Research, Yokohama City University, Yokohama, Kanagawa, Japan; 2 Department of Psychiatry, Yokohama City University, Yokohama, Kanagawa, Japan; 3 Okuda Dental Clinic, Yokohama, Kanagawa, Japan; 4 Division of Genetic Therapeutics, Center for Molecular Medicine, Jichi Medical School, Tochigi-ken, Japan; 5 Laboratory of Experimental Immunology, Cancer and Inflammation Program, National Cancer Institute, National Institutes of Health, Frederick, Maryland, United States of America; Baylor College of Medicine, Jiao Tong University School of Medicine, United States of America

## Abstract

We previously reported on a monoclonal antibody (mAb) that targeted amyloid beta (Aß) protein. Repeated injection of that mAb reduced the accumulation of Aß protein in the brain of human Aß transgenic mice (Tg2576). In the present study, cDNA encoding the heavy and light chains of this mAb were subcloned into an adeno-associated virus type 1 (AAV) vector with a 2A/furin adapter. A single intramuscular injection of 3.0×10^10^ viral genome of these AAV vectors into C57BL/6 mice generated serum anti-Aß Ab levels up to 0.3 mg/ml. Anti-Aß Ab levels in excess of 0.1 mg/ml were maintained for up to 64 weeks. The effect of AAV administration on Aß levels in vivo was examined. A significant decrease in Aß levels in the brain of Tg2576 mice treated at 5 months (prophylactic) or 10 months (therapeutic) of age was observed. These results support the use of AAV vector encoding anti-Aß Ab for the prevention and treatment of Alzheimer's disease.

## Introduction

Alzheimer's disease (AD) is a disorder characterized by a diffuse loss of neurons and the accumulation of amyloid beta (Aß) protein, followed by the production of tau protein or senile plaques in the brain [1]–[2]. Active immunization with Aß peptide was found to reduce the amyloid burden and improve cognitive behavior in murine AD models [3]–[4].

Clinical trials involving peptide immunization were suspended owing to the development of meningioencephalitis in some volunteers vaccinated with Aß peptide [5]–[6]. Clinical studies and autopsy results indicated aseptic meningoencephalitis, presumably induced by the T-cell responses [6]–[8]. Of note, several of the samples obtained from vaccinated patients demonstrated a remarkable reduction in Aß protein levels and senile plaque formation [9]–[10]. These results suggest that if the adverse side effects of such therapy could be avoided, immune mediated elimination of Aß protein could represent a promising therapy for AD.

Based on these observations, the efficacy of intravenous delivery of humanized monoclonal antibodies (mAbs) against Aß was examined [11]–[13]. Despite the widespread reduction in Aß plaques, the passive transfer of mAb reduced AD-like symptoms in only a subset of patients [10]. This observation suggests that neuronal degeneration may occur during the early stages of AD, before the appearance of large Aß aggregates. Thus, it is important to eliminate Aß oligomers at the earliest stages of AD. Previously, we developed a mAb targeting the Aß1–13 peptide. Prophylactic delivery of this mAb or its F(ab')2 fragments to human Aß transgenic mice (Tg2576) effectively prevented the accumulation of Aß protein and plaques [14]. However, Pfeifer et al. [15] reported that anti-Aß mAb treatment could also lead to microhemorrhages in APP23 mice. Moreover, repeated high-dose mAb injections are likely to be very expensive [5], [8].

A potentially safer and more efficacious strategy would be to inject an adeno-associated virus (AAV) that leads to the continuous production of anti-Aß mAb over an extended period. AAV is a nonpathogenic and poorly immunogenic virus. When used as a vector, it can transfer a gene of interest to non-dividing mammalian cells resulting in persistent transgene expression [16].

This work examines the feasibility of using an AAV vector type 1 (AAV vector) modified to encode the anti-Aß Ab to prevent or treat AD in mice. This approach avoids the need to repeatedly administer high doses of mAb. Results suggest that therapy with an Aß mAb-expressing AAV vector greatly reduce Aß accumulation in AD model mice.

## Results

### Production of Ab by cells transfected with the Aß mAb – expressing AAV vector

**Figure 1:**
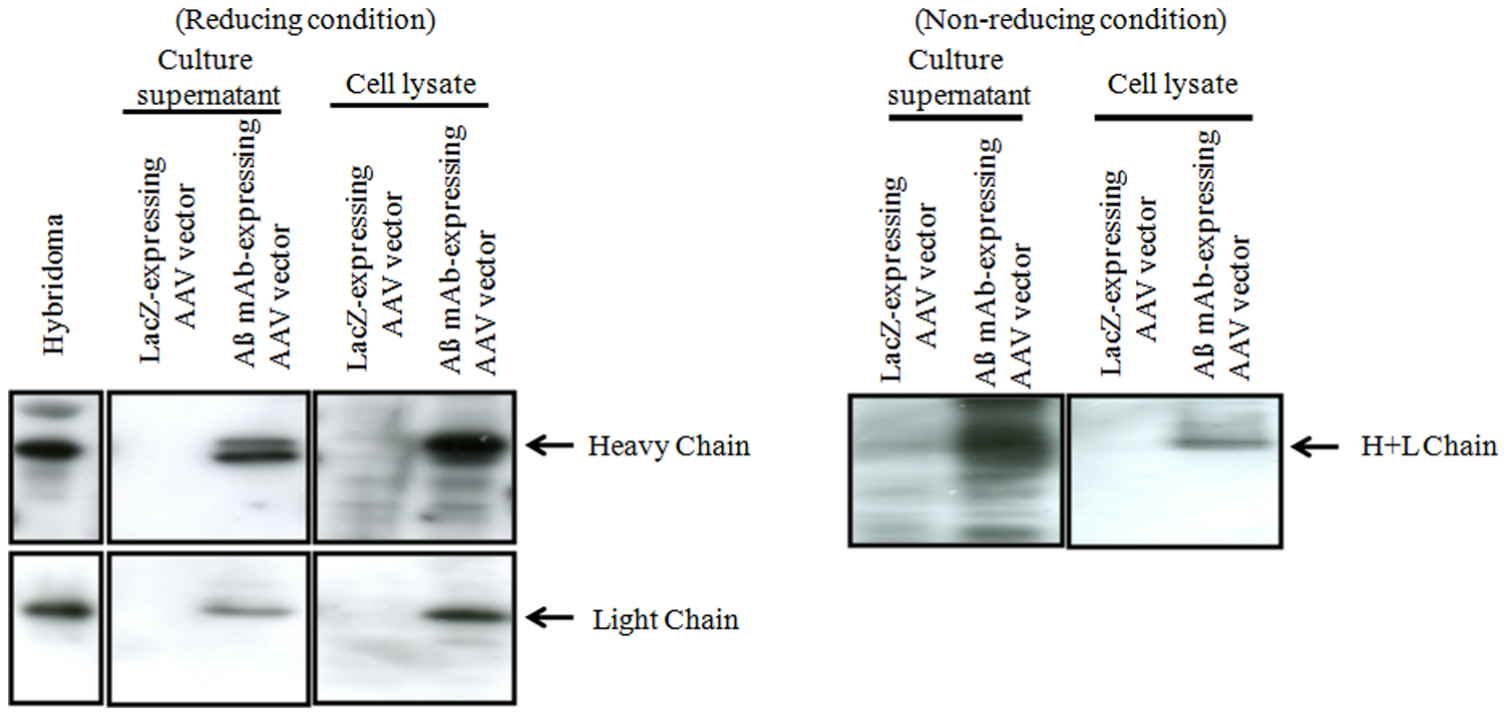
In vitro expression of anti - Aß Abs following the transduction of HEK293 cells with the Aß mAb – expressing AAV vector. Western blots of culture supernatant and cell lysates identify the Ig light and heavy chain (under reducing conditions) and whole Ab (under non-reducing conditions). Cells transfected with a LacZ encoding AAV vector served as negative controls.

We first determined whether the transduction of the new Aß mAb – expressing AAV vector resulted in the production of mAb by HEK293 cells. As shown in Figure 1, we detected Abs in the cell lysates and culture supernatant of the transduced cells. Heavy (H) and Light (L) chains of the appropriate molecular weight were detected. In addition, we detected intact Ab under non – reducing condition. These results indicate that Aß mAb – expressing AAV vector-transduced cells produce proteins with the molecular weight of Abs.

### Binding activity of the Ab produced by AAV vector – transduced cells

**Figure 2:**
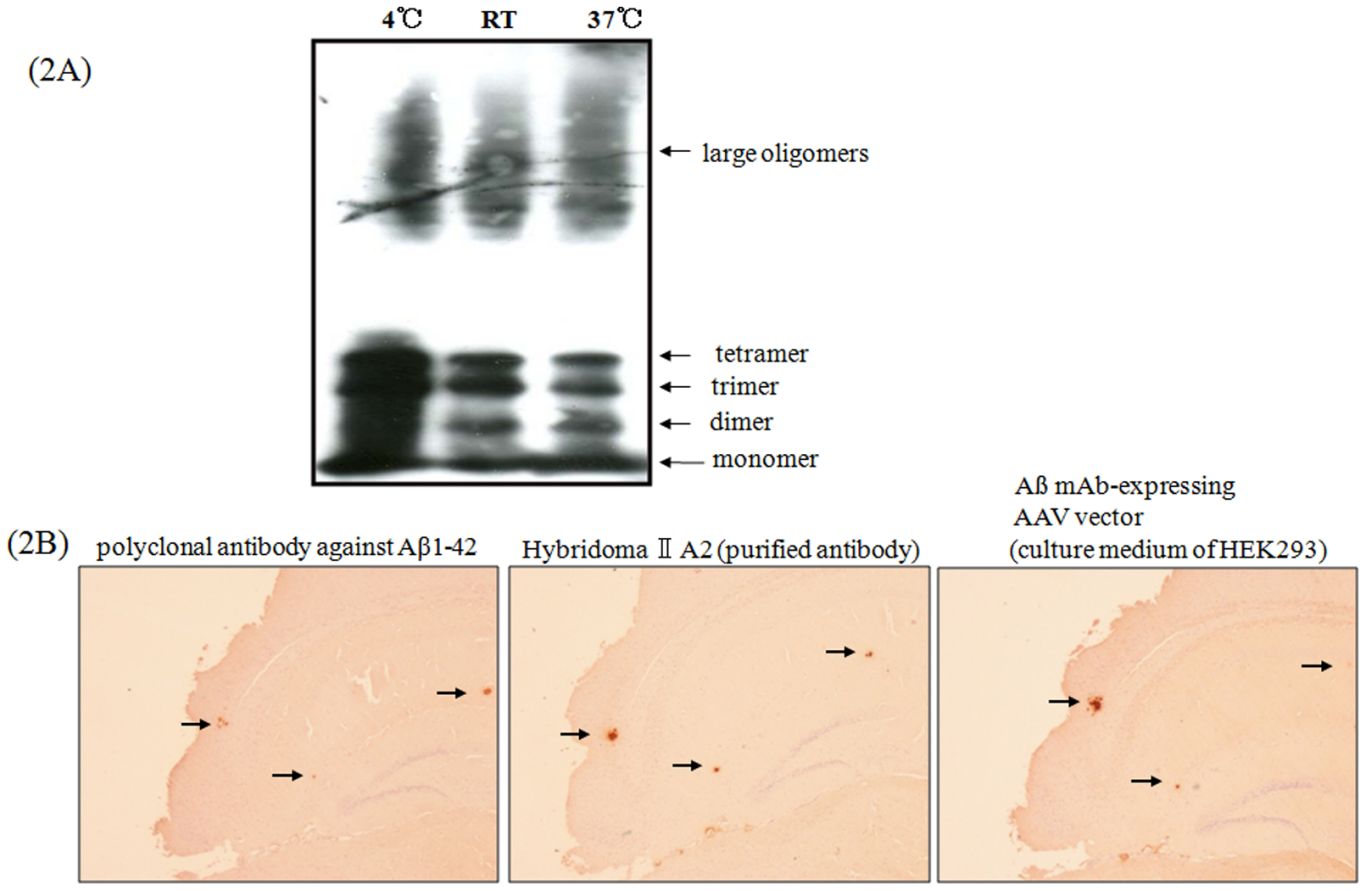
Binding activity of the Ab produced by Aß mAb – expressing AAV vector-transduced cells. A) Binding of Ab from Aß mAb – expressing AAV vector transduced HEK293 cells to synthetic Aß peptides, monomers and oligomers by Western blot. The Ab1-42 peptide was aggregated at 4°C, room temperature (R.T.), or 37°C as described in Materials and Methods and detected by culture supernatant derived from Aß mAb – expressing AAV vector transduced HEK293 cells. B) Anti – Aß Ab derived from transduced HEK293 bound to Aß plaques in 16-month old Tg2576 mice. The specificity of this binding was confirmed by use of polyclonal and monoclonal Abs (see details in Materials and Methods).

We next assessed whether the HEK293 – derived Abs could bind to monomeric Aß protein and oligomerized Aß protein similar to those found in the brain of patients with AD [17]. Results show that culture supernatant derived from Aß mAb-expressing AAV vector-transduced HEK293 cells bound to monomers, dimers, trimers, and tetramers of Aß protein (Figure 2A).

We then analyzed whether Aß mAb – expressing vector-produced Abs bound to Aß aggregates by observing sliced brain sections from Tg2576 mice. Aß aggregates were clearly detected in the brain sections using a polyclonal antibody against Aß1 – 42, anti – Aß1–13 mAb (IIA2), and the culture supernatant from Aß mAb – expressing vector – transduced HEK293 cells (Figure 2B). These results suggest that functional anti-Aß mAb is produced by cells transduced with this Aß mAb – expressing vector.

### Inhibition of hippocampal cell death by Aß aggregates using culture supernatant from AAV-transduced cells

**Figure 3:**
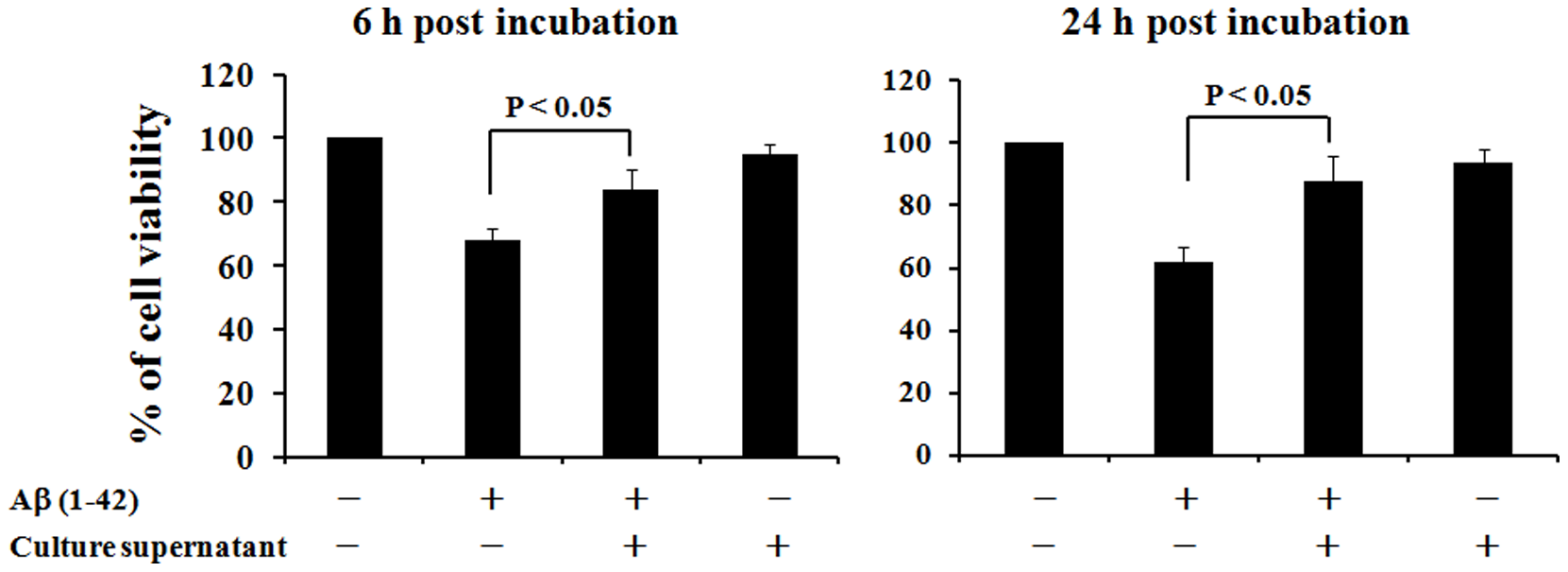
In vitro cytotoxicity inhibition test using primary culture hippocampal cells. The viability of primary hippocampal cells after 6–24 h of culture with 10 uM aggregated Aß protein was examined. The effect of adding culture supernatant from Aß mAb - expressing AAV vector transduced cells was also examined by MTT assay. Data represent the results of 5–8 independently analyzed samples and are presented as mean ± SE.

It is hypothesized that early AD is characterized by the aggregation of Aß protein, and is followed by abnormal tau phosphorylation leading to massive neuronal cell death in the brain. We therefore examined whether the culture supernatant from Aß mAb - expressing AAV vector-transduced cells could inhibit the death of primary hippocampal cells. As shown in Figure 3, synthetic soluble Aß aggregates killed hippocampal cells. This cell death was significantly reduced by the addition of culture supernatant from Aß mAb - expressing vector-transduced cells at 6 h and 24 h after incubation. These results suggest that the culture supernatant of AAV-transduced cells can inhibit the death of primary hippocampal cells.

### Antibody titers of mice infected with the Aß mAb-expressing AAV vector

**Figure 4:**
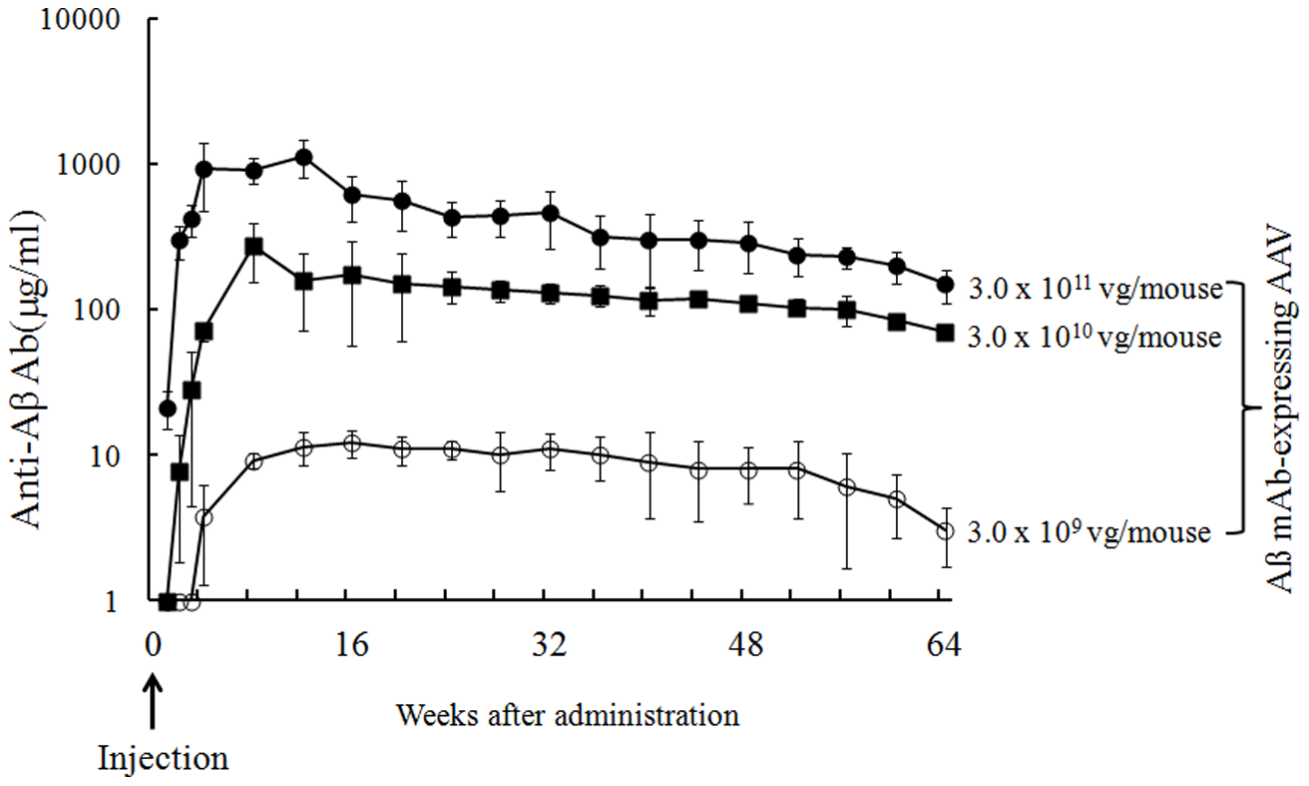
Kinetics of anti-Aß Ab production by mice injected with Aß mAb-expressing AAV. Normal C57BL/6 mice (n = 10/group) received a single intramuscular injection with 3.0×10^9^, 3.0×10^10^, 3.0×10^11^ vg of Aß mAb-expressing AAV or 3.0×10^11^ vg of LacZ-expressing AAV vector. The titer of IgG1 Ab binding to Aß1–42 was detected at indicated time points. Significant difference among groups received 3×10^9^, 3×10^12^ and 3×10^11^ vg of Aß mAb-expressing AAV was observed from 2-week to 64-week after administration (P<0.05). Data are presented as mean ± SE.

Ten weeks old C57BL/6 mice were intramuscularly (i.m.) injected with 3.0×10^9^, 3.0×10^10^ or 3.0×10^11^ viral genome (vg) of the Aß mAb - expressing vector or 3.0×10^11^ vg of LacZ-expressing vector. After administration, serum from these mice was collected monthly and antibody titers were assayed for 64 weeks (Figure 4). Anti-Aß Ab titers peaked approximately 4 weeks after administration and then slowly declined, remaining detectable through 64 weeks of follow up. Ab titers were dose-dependent, with the greatest amount of Ab being present in mice treated with 10^11^ vg of the Aß mAb-expressing AAV vector. In contrast, no Aß-specific Abs were detected in mice injected with the LacZ-expressing AAV vector (data not shown). By 64-week post administration, 0.1 mg/ml of Aβ-specific Ab can be detected in the mice administrated with 3.0×10^10^ vg of the Aß mAb-expressing AAV vector. Considering the safe dosage range of 2.1×10^12^–6.9×10^13^ vg/individual [18] and 2×10^11^–1.8×10^12^ vg/kg [19] in phase 1 clinical trials of intramuscular injection of the recombinant AAV vector, we use the dose of 3.0×10^10^ vg/mouse for further in vivo study, based on the body weight ratio of human beings (60 kg) vs mouse (20 g) and the Ab titer after administration of Aβ mAb-expressing AAV vector (Figure 4).

### Effect of Aß mAb-expressing AAV vector prophylaxis on Tg2576 mice

**Figure 5:**
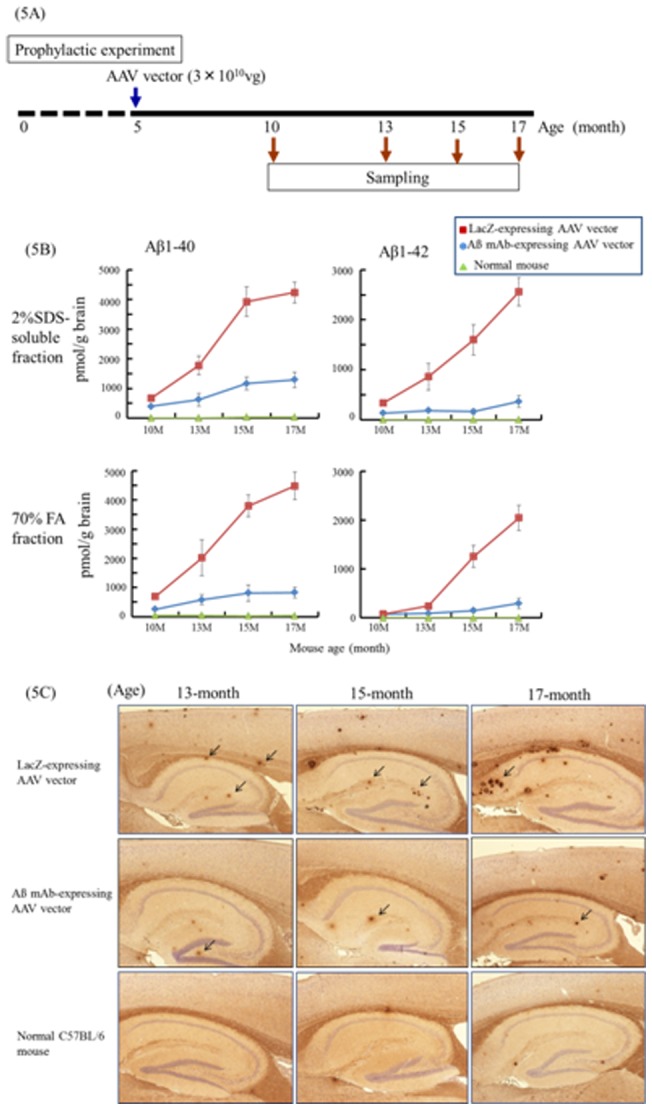
Prophylaxis of AD using Aß mAb-expressing AAV vector. Five-month old Tg2576 mice were injected i.m. with 3×10^10^ Aß mAb-expressing AAV. (A) Scheme of experiment. (B) The amount of Aß protein in brain extracts was determined using anti-Aß Ab-coated ELISA plates at age 10-, 13-, 15- and 17-month old (4–5 mice per time point). C57BL/6 mice were used as controls. Data are presented as mean ± SE. (C) Brain sections from Tg2576 or C57BL/6 mice were examined for Aß deposits by immunohistostaining using rabbit anti-human beta amyloid 1–42 polyclonal antibody.

To determine whether the Aß mAb-expressing AAV vector was able to prevent Tg2576 mice from developing AD, 5-month old animals were injected once with 3.0×10^10^ vg of this vector. Whereas no Aβ protein accumulated in normal mice, there was a statistically significant increase in the amount of Aβ1-40 and Aβ1-42 present in the brain of Tg2576 animals treated with the control LacZ-expressing vector by 10 months of age. The accumulation of this protein continued to rise over time (Figure 5B). By comparison, the amount of Aβ protein present in Tg2576 mice treated with the Aβ mAb-expressing AAV vector was significantly and persistently reduced (Figure 5B).

Serial sagital sections were prepared from the brains of these animals. Aß protein deposits were then visualized immunohistochemically in these sections. Both the size and number of Aβ protein containing deposits increased over time in Tg mice treated with the LacZ-expressing vector. The number of such plaques was significantly reduced among mice treated with the Aß mAb-expressing vector (Figure 5C).

### Effect of Aß mAb-expressing AAV vector treatment on Tg2576 mice

**Figure 6:**
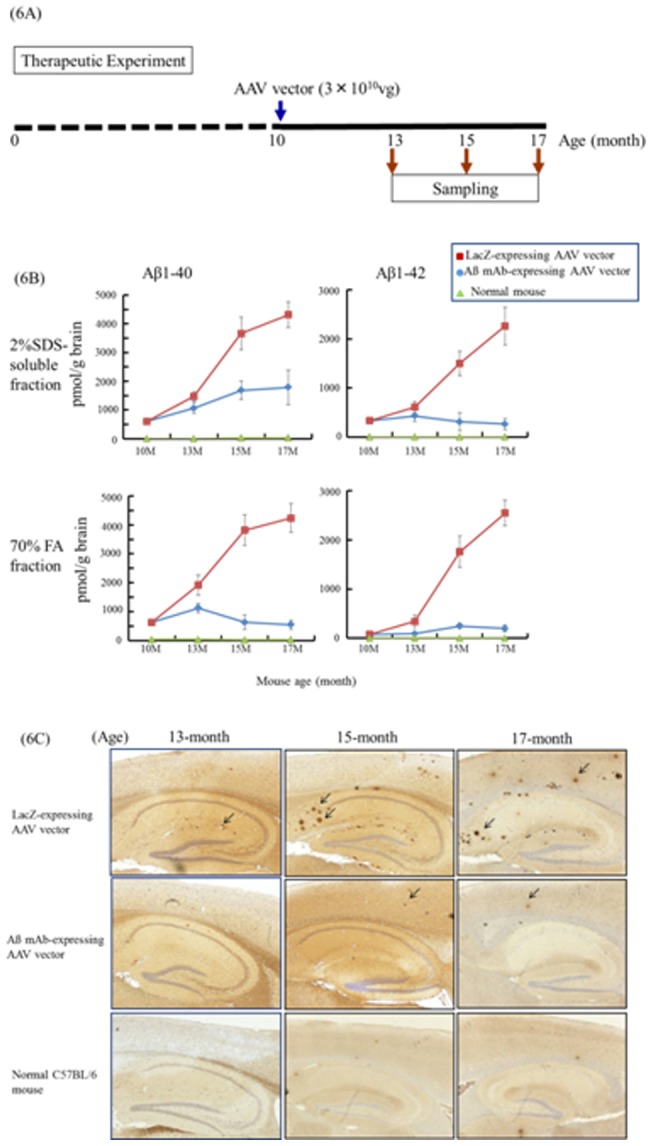
Therapy of AD using Aß mAb-expressing AAV vector. Tem-month old Tg2576 mice were injected i.m. with 3.0×10^10^ vg of the Aß mAb - expressing AAV vector. (A) Scheme of experiment. (B) The amount of Aß protein in brain extracts was determined using anti-Aß Ab-coated ELISA plates at age 10-, 13-, 15- and 17-month old (4–5 mice per time point). C57BL/6 mice were used as controls. Data are presented as mean ± SE. (C) Brain sections from Tg2576 or C57BL/6 mice were examined for Aß deposits by immunohistostaining using rabbit anti-human beta amyloid 1–42 polyclonal antibody.

We finally sought to determine whether the Aß mAb-expressing AAV vector could be used therapeutically. Ten-month old animals were injected with 3.0×10^10^ vg of vector. Aß protein continued to accumulate at 13 months in mice treated with either the LacZ or Aß mAb-expressing AAV vector. However by 15 months (and continuing through 17 months) the size and number of Aß protein containing deposits in the brains of animals treated with the Aß mAb-expressing vector was significantly reduced when compared to LacZ controls (Figure 6B). This divergence was confirmed during the immunohistologic analysis of brain tissue from these animals. Aß protein containing deposits accumulated over time in the LacZ but not the Aß mAb - expressing AAV vector (Figure 6C).

## Discussion

Efforts to treatment AD patients with anti-Aß Abs or through Aß peptide vaccination provided novel insights concerning the pathogenesis of AD and opened new approaches to disease therapy. In an effort to overcome limitations of earlier strategies, the current work examined the effect of delivering an Aß mAb - expressing AAV vector to Tg2576 mice (a murine model of AD). Prophylactic treatment of young (5 months) and therapeutic treatment of older (10 months) animals resulted in a significant and prolonged decrease in the amount of Aß protein accumulating in the brain (Figure 5 and 6).

The AAV vector encoded an anti-Aß mAb that bound to synthetic Aß peptides and to senile plaques present in the brains of Tg2576 mice (Figure 1 and 2). Of interest, a single 3.0×10^10^ vg dose of the Aß mAb-expressing AAV vector resulted in the production of Ab that persisted through the 64-week experimental period (Figure 4). As repeated injection of free anti-Aß mAb can have negative consequences [15], the continuous production of Ab by cells transfected in vivo may provide an ideal method for AD prophylaxis and treatment.

Previous studies investigated the utility of AAV vector for the molecular therapy of Alzheimer's disease. Those vectors encoding antigen, Ab or other factors of potential therapeutic value were examined in various animal models [20]–[31]. These included studies of AAV vector expressing a single-chain variable fragment (scFv) antibody against Aβ protein for AD therapy [22]–[26]. Those studies showed that the scFv fragment had a much shorter serum half-life than whole Ab (7–14 hrs *vs* 20 days) [32], such that the scFv fragment was more suited for intracranial delivery rather than systemic delivery [22]–[26]. Delivery of scFv - expressing AAV vector intracranially reduced/prevented the formation of Aβ brain plaques and improved cognitive function in AD mice [22]–[26], while the delivery method is likely to raise safety issues.

This study examined the effect of treating 5- or 10-month old Tg2576 mice with the Aß mAb - expressing AAV vector. Of importance, a significant decline in the concentration of Aß was found in the brains of both groups of recipient mice (monitored by ELISA and immunohistochemistry). The level of decline was similar in both groups despite the difference in when treatment was initiated (Figure 5 and 6). This may reflect the level of Aß protein being so low in young mice that the effect of therapy cannot be detected until the animals reach 15 months of age. By that time the vector had been active in both treatment groups for a sufficient period to significantly reduce the accumulation of Aß protein

Clinical trials showed that vaccination of AD patients with an Aß peptide reduced the deposition of Aß in some individuals [10]. Unfortunately, this treatment also led to the development of cerebroencephalitis in some patients, a side effect so severe that further development of this type of therapy was abandoned [11], [14], [33]. An alternative approach involved the intravenous administration of Abs against Aß peptide [5], [8]. While effective at reducing the accumulation of Aß aggregates, the injection of anti-Aß Ab resulted in a high incidence of cerebral microhemorrhages [15]. Approximately 30% of AD patients have cerebral amyloid angiopathy (CAA)-associated microhemorrhages [34]. Shroeter et al. [35] reported that anti-Aß Ab treatment of 12-month old AD model mice resulted in a dose-dependent reduction of the occurrence of CAA after treatment. In older model mice, CAA is known to be relatively abundant [36]–[37]; however, the injection of a small amount of anti-Aß mAb did not increase the incidence of microhemorrhages [36]–[37], indicating that long-lasting expression of anti-Aß mAb by AAV vector may have some advantages in reducing the incidence of microhemorrhages. Many reports have explored the T-cell response after virus or non-virus-based vaccine against Alzheimer's. The T-cell response is very low or undetectable [38]–[40]. In this study, we have not explored the T-cell response, because our AAV vector expresses anti-Aβ-antibody, but not an antigen. Furthermore, we histologically evaluated the appearance of microhemorrhages and inflammation of brain in the mice treated with the Aß mAb-expressing AAV vector. No evidence of this adverse side effect was observed even in 17-month old Tg2576 mice (data not shown).

Recent reports suggest that mAb therapy is effective only in AD patients possessing e4/e4 proteins [12], [41]. In this context, ApoE4 (+) individuals develop AD more often than ApoE4 (−) individuals [42]–[43]. Such observations help inform the design of trials with AAV vectors intended for human use, as it facilitates the identification of individuals at high risk for developing AD. In addition, previous studies documented that Aß oligomers are toxic to neuronal cells [44]–[45]. That finding is consistent with current results showing that aggregated Aß proteins are toxic to primary culture neuronal cells and that anti-Aß mAbs prevent this toxicity (Figure 3).

We hypothesize that the ongoing accumulation of Aß aggregates is responsible for widespread neuronal cell degeneration and the subsequent dementia characteristic of AD. We believe that the failure of clinical trials involving anti-Aß mAb may reflect the late initiation of such treatment as it is important to eliminate Aß oligomers during the early stages of AD. Evidence that ApoE4 and other factors can predict individuals at high risk [34] lead us to recommend clinical trials of Aß mAb - expressing AAV vector commence at an early age for prophylaxis of AD. Current results show that the production of anti-Aß mAbs persists for an extended period (Figure 4). Thus, a single early treatment may yield long term clinical benefit. Further study that includes behavioral and memory testing combined with even longer follow should help clarify the value of AAV vector mediated in the prophylaxis and/or therapy of AD treatment.

Taken together, we constructed an anti-Aβ Ab-expressing AAV vector. A single intramuscular injection of the vector generated high serum anti-Aß Ab level for up to 64 weeks, and significantly decreased Aß levels in the brain of AD model mice treated at 5 months (prophylactic) or 10 months (therapeutic) of age. Our present results clearly demonstrated that the Aß mAb-expressing AAV vector may be of prophylactic and/or therapeutic value for AD treatment.

## Materials and Methods

### Ethics Statement

All animal work has been conducted according to relevant Japan and international guidelines. All experimental procedures were carried out in accordance with the Administrative Panel on Laboratory Animal Care (APLAC) protocol and the institutional guidelines set by Yokohama City University and Chyoju Medical Institute. The protocols used in this study were proved by Institutional Animal Care and Use Committee (IACUC)/Ethics committee of Yokohama City University (No. 0741, 0875 and 0974) and Chyoju Medical Institute (No. B-22).

All animal work has been conducted according to relevant U.S. and international guidelines. Specifically, all experimental procedures were carried out in accordance with the Administrative Panel on Laboratory Animal Care (APLAC) protocol and the institutional guidelines set by the Veterinary Service Center at Stanford University (Animal Welfare Assurance A3213-01 and USDA License 93-4R-00). Stanford APLAC and institutional guidelines are in compliance with the U.S. Public Health Service Policy on Humane Care and Use of Laboratory Animals. The Stanford APLAC approved the animal protocol associated with the work described in this publication.

### Genes of mAbs against Aß

A hybridoma producing anti-Aß1–13 mAb (IIA2) [14] was cultured in KBM 450 medium (Kohjin Bio Co., Ltd., Saitama, Japan) in the absence of fetal bovine serum (FBS). The antibody was concentrated and partially purified from culture supernatant by ammonium sulfate precipitation and was used for the experiments as a positive control.

### Aß peptide synthesis and aggregated Aß or oligomer formation

The Aß1-42 peptide used in these studies was produced by chemical synthesis (American Peptide, Sunnyvale, CA, USA). Reverse - phase high performance liquid chromatography showed that the synthesized peptide has >95% purity, and mass spectrometry analysis verified the molecular mass. Oligomer formation was done using the method described previously (Stine et al., 2003). Briefly, Aß oligomers were prepared by diluting 5 mM Aß1–42 in Me2SO to 100 μM in ice-cold cell culture supernatant (phenol red - free Ham's F-12; BioSource, CA, USA), immediately vortexing for 30 s, and incubating at 4°C, room temperature or 37°C for 24 h. The aggregated Aß or oligomer solution was used for the Western blotting analysis, as well as cytotoxic tests of neural cells.

### Construction of an expression vector for the anti-Aß mAb gene

Total RNA was extracted from a hybridoma producing anti-Aß1–13 mAb (IIA2) [14] using TRIzol Reagent (Gibco BRL, Grand Island, NY, USA). Full-length heavy (H) chain and light (L) chain cDNA was transcribed with 5′-RACE primer and 3′-RACE primer using BD SMART RACE cDNA Amplification Kit (Clontech, Mountain View, CA, USA) according to the manufacturer's instructions. H chain cDNA was amplified with sense primer (5′ CGG GGT ACC ATG GGC AGG CTT ACT TCT TC 3′) and antisense primer (5′ CCC AAG CTT TTT ACC AGG AGA GTG GGA GA 3′). L chain cDNA was amplified with sense primer (5′ CCG GAA TTC ATG GAG ACA GAC ACA CTC CT 3′) and antisense primer (5′ ATA AGA ATG CGG CCG CA G TCG ACG CTA ACA CTC ATT CCT GTT GA 3′). The Furin 2A fragment [46]–[47] from the foot and mouth disease virus was synthesized with complementary oligo (5′ CCC AAG CTT CGC GCC AAG CGC GCC CCC GT 3′ and 5′ CCG GAA TTC GGG GCC GGG GTT GGA CTC CA 3′). The H chain–Furin 2A–L chain fusion fragment was subcloned into proviral plasmid pW1 controlled by the CMV promoter. The AAV vectors were prepared by the previously described three - plasmid transfection adenovirus-free protocol [16], [48]. Briefly, 60% confluent human embryonic kidney 293 (HEK293) cells were maintained in Dulbecco's modified Eagle's medium – nutrient mixture F-12 (1∶1) (DMEM/F-12; GIBCOBRL, New York, NY) supplemented with 10% fetal bovine serum (FBS). Cells were cultured at 37°C in an atmosphere of 5% CO2 in air. Subconfluent HEK293 cells were co-transfected by the calcium phosphate co-precipitation method with the AAV shuttle plasmid pW1 (containing LacZ or antibody heavy chain-F2A-light chain), the AAV-1 chimeric helper plasmid p1RepCap (provided by Dr. James M. Wilson, University of Pennsylvania, Philadelphia, PA, USA), and the adenoviral helper plasmid pAdeno (provided by Avigen, Inc., Alamada, CA, USA). After 48 h, the cells were harvested and lysed in Tris buffer (10 mM Tris-HCl, 150 mM NaCl, pH 8.0) by three cycles of freezing and thawing. One round of sucrose precipitation and two rounds of CsCl density-gradient ultracentrifugation were sufficient to isolate the AAV vector from the lysates. The vector titer was determined by quantitative PCR and presented as vg.

### Western blot analysis

To confirm the expression of the Ab proteins, HEK293 cells were transduced with the AAV vector encoding the mAb genes (Aß mAb-expressing vector) in a 6-well plate. After transduction of AAV vector for 2 h, the cells were washed twice with PBS and cultured with Ex-CELL CD CHO serum-free medium (Life Technologies Japan Ltd, Tokyo, Japan) for another 2 days. Then, antibody protein was detected in culture supernatant and cell lysates with Western blot. The cells were washed with PBS and lysed with 0.1 M Tris-HCl (pH 7.8) and 0.125% Nonidet P-40 2 days after transduction. The cell lysates were mixed with an equal volume of 2× SDS buffer (125 mM Tris-HCl [pH 6.8], 4% SDS) with 100 mM of DTT (reducing condition) or without DTT (non-reducing condition) and boiled for 10 min. The cell lysates were loaded on an 8% polyacrylamide gel and transferred to a Hybond ECL nitrocellulose membrane. After rinsing with PBS, the membrane was probed with HRP-labeled goat anti-mouse IgG1 or IgκAbs (Ig; ICN Pharmaceuticals Inc., Solon, OH, USA). The protein was detected using the ECL Plus Western Blotting Detection System (Amersham Pharmacia Biotech, Uppsala, Sweden).

### Animals and administration

Heterozygous Tg2576 mice were obtained from Taconic Farms Inc. (Germantown, NY, USA) [14]. Normal C57BL/6 female mice were purchased from Japan SLC Inc. (Hamamatsu, Japan). The mice were housed in the animal centers located at Yokohama City University and Chyoju Medical Institute, and maintained on a 12-h day–night cycle. Five-month and 10-month old mice were used for prophylactic and therapeutic experiments, respectively. The mice were received a single injection in quadriceps muscles with a dose of 3.0×10^10^ vg of the Aß mAb-expressing vector. We used 20–25 mice in each group. The mice for prophylactic experiments were sacrificed 5, 8, 10 and 12 months after Aß mAb - expressing vector administration and the mice for therapeutic experiments were sacrificed 0, 3, 5 and 7 months after Aß mAb-expressing vector administration. To explore the magnitude and duration of antibody expression, we injected 3.0×10^9^, 3.0×10^10^ and 3.0×10^11^ vg of Aß mAb-expressing vector or 3.0×10^11^ vg of AAV vector expressing LacZ gene (LacZ-expressing vector) to mouse quadriceps muscles, and blood was collected at indicated time points for Aβ-specific antibody detection. The animal experiments were approved by the Animal Ethical Committees of Yokohama City University School of Medicine and and Chyoju Medical Institute.

### Enzyme-linked immunosorbent assay (ELISA) for Ab titers

ELISA was performed as described previously [14]. Briefly, 96-well microtiter plates were coated with 40 μg/ml of Aß1-42 peptide in 0.15 M phosphate-buffered saline (PBS). The wells were rinsed with 0.15 M PBS and then blocked with 3% FBS in 0.15 M PBS for 1 h. Appropriately diluted mAb as well as the serum from AAV vector-administered mouse were incubated on antigen-coated plates for 6 h at 4°C. Then, wells were rinsed with 0.15 M PBS and the bound Abs were detected using HRP-coupled goat anti-mouse IgG1 (Pierce Chemical Co., Rockford, IL, USA). We detected IgG1 titer rather than total IgG titer, because transgene of the AAV vector was isolated from the mouse IgG1-secreting hybridoma (IIA2). The anti-Aß1–13 mAb (IIA2) purified from the hybridoma was used as a standard control.

### Brain sample preparation for histochemical studies

Brains were removed and divided sagittally along the interhemispheric fissure. The right hemisphere was dissected from the cerebella. Brain samples were snap frozen in large test tubes containing n-hexane, immersed in a dry ice/acetone mixture, and stored at −80°C until processing. The left hemisphere was fixed with formalin and then embedded in paraffin for histochemical studies. To test whether Aß mAb-expressing vector-produced antibody can be used for immunostaining against human Aß, Culture supernatant of Aß mAb - expressing vector-transduced HEK293 cells was used as a first antibody and HRP-Goat ant-mouse IgG1 antibody used as a second antibody. Rabbit anti-human beta amyloid (1–42) antibody (Genetex, Inc., Irvine, CA) and mAb purified from hybridoma IIA2 were used as controls. Paraffin samples from AAV vector administrated mice were stained with the rabbit anti-human beta amyloid (1–42) antibody.

### ELISA to measure Aß protein levels in the brain

The frozen left cerebra were obtained from each mouse and homogenized with a homogenizer in Tris-buffered saline buffer (TBS, 50 mM Tris, 150 mM NaCl, pH 7.6) containing protease inhibitor cocktail (Nacalai, San Diego, CA, USA) with 20 μg/ml pepstatin A, then centrifuged at 100,000 *g* for 1 h at 4°C using an Optima TLX ultracentrifuge (Beckman Coulter Inc., Fullerton, CA, USA). The pellets were homogenized in TBS buffer containing 2% SDS and protease inhibitor cocktail (Nacalai, San Diego, CA, USA) following incubation at 37°C for 15 min. Then the solution was centrifuged again at 100,000 *g* for 1 h at 25°C. The supernatant and pellet correspond to the soluble (2% SDS soluble fraction) and insoluble fraction, respectively.

The insoluble fraction was washed, then extracted again with 70% formic acid and centrifuged at 100,000 *g* for 1 h. The supernatants of 70% formic acid extracts were neutralized with 1 M Tris-HCl, pH 8.0 at a dilution of 1∶20 (70% FA fraction). The dissolved samples of Aß1–42 or Aß1–40 protein were quantified using Human Amyloid ß (N3pE-42) Kit and Human Amyloid ß (N3pE-40) assay kit, respectively (IBL Co., Ltd., Gunma, Japan). The values obtained were corrected with the wet weight of each brain hemisphere sample and expressed as pmol/g brain.

### Cytotoxicity inhibition test by Abs from AAV-transduced cells using primary culture hippocampal cell

Hippocampal cells were collected from 15-day-old fetal mouse brains and gently minced. The samples were incubated at 37°C for 5 min in 9 ml of 0.15 M PBS and 1 ml of 2.5% trypsin, followed by addition of 1 ml 0.5% trypsin inhibitor on ice. The cells were washed twice with 0.15 M PBS and resuspended in media stock (MS) supplemented with 20% FBS, 10 ng/ml epidermal growth factor, 50 IU/ml penicillin, and 50 μg/mL streptomycin. MS is composed of modified Eagle's medium supplemented with 2 mM glutamine and 20 mM glucose [49].

We reacted 5.0×10^4^ primary culture hippocampal cells in 100 μl MS per well with each concentration of aggregated Aß solution with or without Aß mAb - expressing vector-transduced culture supernatant in a humidified atmosphere (37°C, 5% CO2). After 6 and 24 h incubation, 10 μL 3-[4,5-dimethylthiazol-2-yl]-2,5-diphenyl tetrazolium bromide (MTT) reagent (final concentration 0.5 mg/ml) was added to each well. The microplate was incubated for 4 h in a humidified atmosphere [50]. Then, 100 μl of the solubilization solution was added into each well to solubilize the purple formazan crystals, and the absorbance of the samples was measured using an ELISA microplate reader [51].

### Data analysis

All values were expressed as the mean ± standard error (SE). Statistical analysis (Student's t-test) of the experimental data and controls was conducted using two-way factorial analysis of variance. Significance was defined as P<0.05 for statistical analysis using all time points in each group.
